# Mechanical Properties and Feasibility of GFRP from Decommissioned Large-Scale Wind Turbine Blades for Wave Energy Converter: A Case Study

**DOI:** 10.3390/polym18070892

**Published:** 2026-04-06

**Authors:** Yan-Wen Li, Jin-Sheng Lai, Bin-Zhen Zhou, Li Cheng

**Affiliations:** 1 State Key Laboratory of Subtropical Building and Urban Science, South China University of Technology, 381 Wushan Road, Guangzhou 510641, China; reallai@163.com; 2 School of Civil and Transportation Engineering, South China University of Technology, Guangzhou 510640, China; 3 School of Marine Science and Engineering, South China University of Technology, Guangzhou 510640, China; zhoubinzhen@scut.edu.cn (B.-Z.Z.); licheng_scut@126.com (L.C.)

**Keywords:** decommissioned wind turbine blades, mechanical properties of GFRP, graded reuse of GFRP, blade-repurposed wave energy converter, carbon footprint assessment

## Abstract

Repurposing decommissioned wind turbine blades provides a vital pathway to mitigate carbon emissions, yet the escalating volume of large-scale waste poses a severe environmental challenge. Recognizing the limitation that existing research focuses predominantly on small-scale legacy blades, this study addresses this gap by assessing the mechanical properties and microstructure of a 54-m (2.0 MW) blade decommissioned due to repowering after 10 years of service. GFRP samples extracted from the root, mid-span, and tip were investigated using X-ray computed tomography and a comprehensive suite of mechanical tests. The investigation confirmed a low internal porosity (~1.2%) without service-induced macroscopic interfacial cracking, alongside superior residual performance, exemplified by a tensile strength of 849.5 MPa at the root. Statistical analysis employing ANOVA revealed significant spatial variations, supporting a graded reuse strategy: roots with superior tensile strengths for critical members, mid-spans for axial compression, and tips as a reliable property baseline for general reuse, while Weibull analysis verified the statistical reliability required for structural design. Based on these superior residual properties, a raft-type wave energy converter utilizing repurposed blade segments was proposed. A comparative carbon footprint assessment revealed that this blade-repurposed WEC achieved a 71.5% reduction in carbon emissions and a 37.4% reduction in structural mass compared to conventional steel counterparts. These findings substantiate the viability of large-scale DWTBs as high-value resources for decarbonizing marine infrastructure within a circular economy.

## 1. Introduction

The rapid expansion of the wind energy sector [1,2] has caused a looming environmental challenge: the volume of decommissioned wind turbine waste is projected to reach 43 million tonnes globally by 2050 [3]. This surge results from both the End-of-Life (EOL) of aging turbines, typically reaching the end of their 20-to-25-year service life, and the early retirement of units due to technological upgrades [4]. While metal and concrete components from towers and foundations have established recycling pathways [5,6], wind turbine blades pose a critical management issue because of their complex material system. Typically comprising reinforcing fibers, matrix resins, core materials, and auxiliary materials [7], blades are primarily composed of about 80 wt% thermoset-based glass fiber-reinforced polymers (GFRP) [8]. Due to the irreversible cross-linking of the thermoset matrix, these composites are non-degradable and difficult to recycle using traditional methods [9,10,11]. However, GFRP’s high strength, corrosion resistance, and low density [12,13] offer significant potential for high-value resource utilization.

Guided by circular economy principles and the waste management hierarchy, the end-of-life (EOL) treatment of decommissioned wind turbine blades (WTBs) is systematically categorized into three primary strategies: Recycling, Reuse, and Repurposing [5,14]. Recycling encompasses thermal (pyrolysis) [15,16], chemical (solvolysis) [17,18], mechanical [9,19], and emerging freeze-thaw [20] pathways; however, these approaches often face limitations, as thermal and chemical recycling entail high energy demands, while mechanical recycling yields low-value fillers that fail to harness the materials’ original structural strength [2]. Consequently, sustainable management prioritizes Reuse, which focuses on lifetime extension through structural monitoring, maintenance, and repair [6,21], and Repurposing (structural reuse). Particularly concerning greenhouse gas (GHG) emissions, repurposing emerges as a superior pathway [22], as it preserves the macro-structural integrity of the composites while achieving substantial carbon avoidance and directly displaces energy-intensive virgin materials [23].

**Figure 1:**
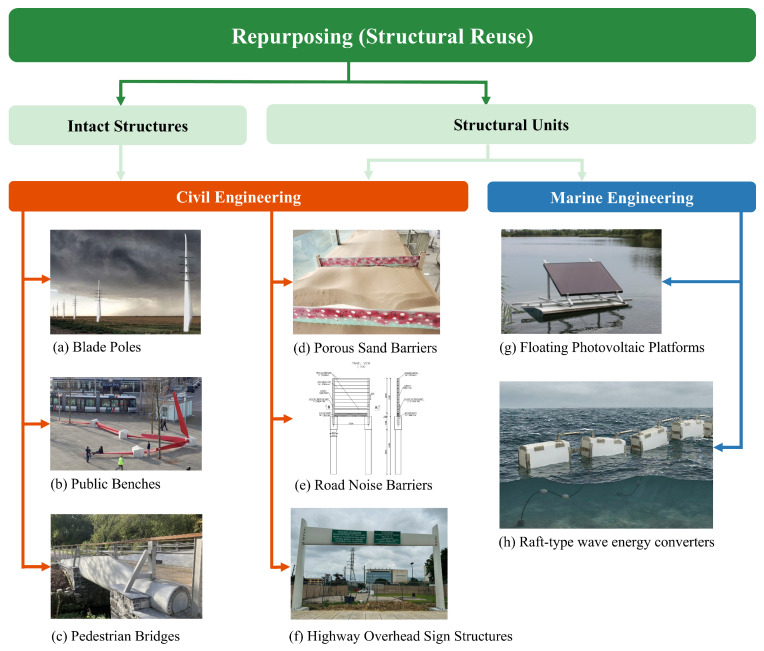
Structural reuse strategy classification of decommissioned wind turbine blades: (**a**) Blade Poles [34], (**b**) Public Benches [35], (**c**) Pedestrian Bridges [24], (**d**) Porous Sand Barriers [25], (**e**) Road Noise Barriers [26], (**f**) Highway Overhead Sign Structures [28], (**g**) Floating Photovoltaic Platforms [36], (**h**) Raft-type wave energy converters (This research).

Recent studies have proposed a dual-scale framework for structural repurposing, as shown in Figure 1. The macro-level strategy involves utilizing the blade as an intact structure [2]. Within civil engineering, this whole-blade utilization is ideally suited for substantial load-bearing constructions, such as pedestrian bridges [24,25]. However, the direct deployment of entire blades frequently encounters severe practical hurdles, including transportation bottlenecks, strict regulatory codes, and spatial limitations. To bypass these barriers and cope with the ever-increasing dimensions of modern WTBs, researchers have highlighted a secondary, more versatile tier: segmenting the blades into structural units [2,9]. This component-level disassembly significantly enhances the scalability of mechanical recycling, facilitating the integration of WTBs into various terrestrial public works, such as porous sand barriers [26], noise barriers [27], and highway overhead sign structures [28]. Despite these advancements in civil infrastructure, repurposing WTBs for marine engineering remains remarkably scarce, with current efforts mostly confined to preliminary applications like floating photovoltaic platforms [6]. Furthermore, the inherent high strength and exceptional seawater corrosion resistance of glass fiber-reinforced polymers (GFRP) well align with the stringent material requirements of wave energy converters operating in harsh marine environments. Therefore, transitioning repurposing strategies from terrestrial infrastructure to innovative marine engineering, specifically by transforming DWTBs into a raft-type wave energy converter, represents a highly promising and under-investigated research frontier.

**Figure 2:**
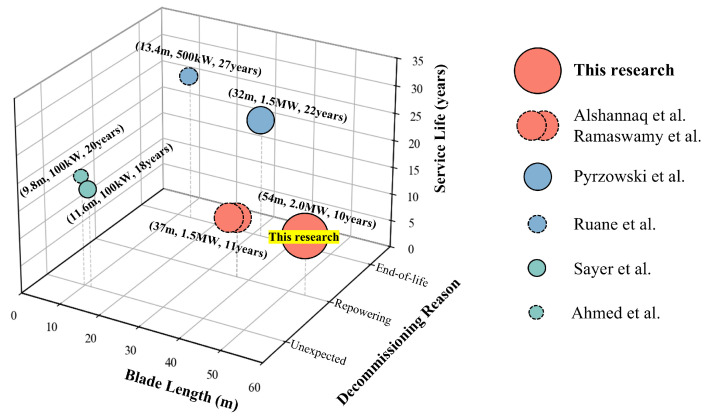
Distribution diagram of mechanical properties for decommissioned blades. data are sourced from Alshannaq et al. [32], Ramaswamy et al. [33], Pyrzowski et al. [31], Ruane et al. [24], Sayer et al. [29], and Ahmed et al. [30].

However, safe repurposing relies on accurate mechanical assessment. Figure 2 visually illustrates the distribution of existing literature on the mechanical properties of decommissioned blades. Currently, research on the mechanical properties and reuse of decommissioned wind turbine blades primarily focuses on four core directions. Firstly, earliest pioneering works, mainly by Sayer et al. [29] in the aspect of service evaluation and real-world load benchmark comparison, utilized actual wind condition data from meteorological stations to directly compare decommissioned blades with early original laboratory test data, in order to assess the actual consumption of the material’s fatigue life and stiffness caused by real-world load spectra. Secondly, in the field of environmental coupled damage assessment, Ahmed et al. [30] focused on exploring the coupled effects of varying temperature environments (−10 °C to +50 °C) and fiber orientations on the residual tensile and compressive properties of aging blades. Thirdly, for the TRIAX laminates used in aerodynamic shells, Pyrzowski et al. [31] concentrated on material parameter reconstruction and theoretical stiffness identification. Finally, in recent years, civil engineering structural applications and statistical design have emerged as the mainstream research direction in this field, represented by the studies of scholars such as Ruane et al. [24], Alshannaq et al. [32], and Ramaswamy et al. [33]. They are dedicated to directly repurposing the spar cap materials of decommissioned blades into secondary infrastructure load-bearing components, such as pedestrian bridges, transmission poles, and highway overhead sign structures, based on mechanical performance testing, while strongly emphasizing the utilization of mathematical models like the Weibull distribution to extract statistically significant safety characteristic values, providing reliable lower-bound data support for conservative safety designs in civil engineering.

In spite of these valuable contributions, current studies primarily focus on small-scale blades (<15 m, <1 MW) with long service lives (approximately 20 years) [29,30]. Although Alshannaq et al. [32] and Pyrzowski et al. [31] advanced the field by investigating 37 m (1.5 MW) blades decommissioned due to repowering, the continuous upscaling of wind turbines dictates that future retired blades will inevitably be larger and may feature different material compositions or structural designs. Within the scope of blades retired early due to technical upgrades, existing studies are limited to the 37 m scale. Therefore, a distinct scientific data gap exists regarding the mechanical behavior of larger, 50-m-scale blades. Given the continuous upscaling of wind turbines, there is no theoretical upper limit to the size of decommissioned blades; however, the 54-m blade investigated in this study represents the largest scale of decommissioned blades currently available for comprehensive mechanical evaluation, significantly exceeding the dimensions reported in existing literature. Understanding the residual performance of these large-scale blades is crucial for future recycling efforts, yet empirical data remain exceedingly scarce.

Furthermore, while conventional destructive testing provides essential mechanical data, existing characterization methodologies predominantly rely on localized 2D microscopic observations. Integrating these tests with high-resolution 3D non-destructive X-ray CT imaging can offer a more multi-dimensional perspective on the internal integrity of these thick composites. Concurrently, for blades longer than 20 m, the sampling has generally been restricted to singular regions, leaving the spatial distribution of mechanical properties along the full span largely unexplored. Finally, although current repurposing strategies include basic civil infrastructure, there remains a significant opportunity for cross-sector, high-value innovations, such as marine wave energy converters (WECs), that fully exploit the GFRP’s inherent durability and corrosion resistance.

To bridge those scientific gaps, this study investigates a 54 m, 2.0 MW blade, which was decommissioned due to repowering after 10 years of service. This specific blade was selected as it is the longest decommissioned blade of significant research value that could be practically sourced, considering the combined constraints of service location, decommissioning context, and transportation. By evaluating this relatively younger material with potentially higher residual strength, this study provides rare empirical data on a utility-scale (>50 m) blade. This study employs characterization methods distinct from current literature to explicitly analyze the mechanical properties and microstructures of GFRP from this large-scale 2.0 MW blade. By combining non-destructive X-ray computed tomography (CT) with destructive ignition loss (IL) tests, this work characterizes the microstructure of GFRP. This approach provides a scientific basis for evaluating the feasibility and reliability of reusing residual performance in secondary engineering applications for megawatt-scale wind turbine blades. Specifically, the study encompasses the following aspects: (i) characterizing the material at the microscopic level; (ii) determining the mechanical properties of GFRP across various blade regions, analyzing failure behavior under different load modes, and conducting a rigorous statistical analysis of the data; and (iii) proposing a cross-sector repurposing concept based on the blades’ performance advantages by designing a blade-repurposed raft-type wave energy converter and comparing its carbon emissions with those of traditional steel counterparts. Collectively, these efforts provide critical scientific guidance for the safe and efficient second-life applications of GFRP from DWTBs while establishing a solid foundation for advancing repurposing strategies and assessing the lifespan of composite materials within a circular economy framework.

## 2. Materials and Methods

A single blade was selected from an operational wind farm to evaluate the mechanical properties of its GFRP composite. After samples were extracted and prepared from the blade, mechanical testing was performed strictly in accordance with relevant ASTM and ISO standards, encompassing a full range of tests: tensile, 0° and 90° compression, three-point flexural, short beam shear, interlaminar shear, and in-plane shear. To validate the relationship between the measured mechanical properties and internal microstructural features, including fiber distribution, orientation, volume fraction, and defects, X-ray CT and the ignition loss method were employed.

### 2.1. Material Preparation

#### 2.1.1. Decommissioned Blade Source

The selected blade, Model DT112-2000/A7 (Nantong Dongtai New Energy Equipment Co., Ltd., Nantong, China) from a 2MW-XE112-2000 generator (XEMC-Darwind, Xiangtan, China), featured nominal specifications of 54 m in length and a total weight of 11,875 kg. The blade is primarily composed of E-glass fiber and an epoxy resin system, manufactured using the vacuum infusion process. This blade accumulated approximately 10 years of service life at a wind farm located in Yongzhou, Hunan Province, China (260.5 m above sea level). The operational environment is characterized by severe weather exposure, featuring frequent rainstorms and high lightning activity during spring and summer. Crucially, the blade was retired due to a technical upgrade of the turbine, thus presenting an ideal specimen for assessing the residual mechanical properties of naturally aged GFRP.

#### 2.1.2. GFRP Sample Preparation

**Figure 3:**
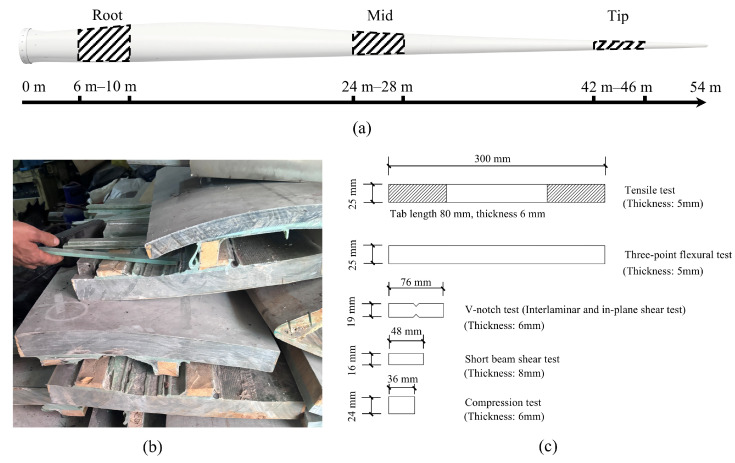
Preparation of GFRP samples. (**a**) Sampling regions in the blade; (**b**) Post-cutting blades; (**c**) Geometric parameters.

The sample selection strategy was governed by both scientific rigor (accounting for property distribution along the span) and logistical constraints (minimizing transportation and processing costs). Spatially, GFRP samples were extracted on-site from three distinct regions along the 54 m blade: root, middle, and tip, as shown in Figure 3a. Each sample measured 4 m in length and was spaced 18 m apart. Regarding fiber orientation, previous studies [31,33] indicate that GFRP transverse properties are significantly inferior to longitudinal ones. Since both blades and the targeted civil reuse applications rely primarily on longitudinal fibers for axial and bending loads, this study focused exclusively on longitudinal samples to capture the primary load-bearing capacity. Test samples were machined to the geometries specified in Figure 3c.

### 2.2. Characterization Methods

#### 2.2.1. X-Ray Computed Tomography

X-ray CT enables high-resolution 3D non-destructive characterization of anisotropic composites, offering superior visualization of internal defects and fiber architecture in GFRP compared to other methods [37,38]. Furthermore, CT has also been proposed as a potential approach for assessing the reusability of DWTBs [39]. In this study, a high-resolution CT system (METROTOM 800, 225 kV; ZEISS Industrial Quality Solutions, Oberkochen, Germany) was employed to characterize GFRP morphology. Imaging relies on the linear attenuation coefficient *μ*, which depends on material density *ρ* and atomic number *Z*, and is mathematically expressed by Equation (1) [40]:

(1) $$\mu \left(x,y,z\right)=K\rho \frac{Z^{4}}{E^{3}}$$

where: *μ* is the linear attenuation coefficient, *K* is a constant, *ρ* is the material density, *Z* is the atomic number, and *E* is the energy of the incident photon (experimentally controlled).

**Figure 4:**
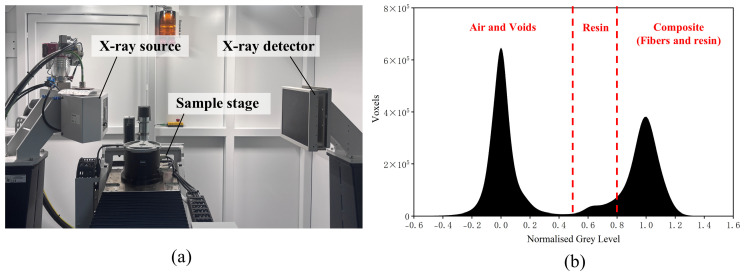
Microstructural characterization via X-ray CT. (**a**) CT internal components; (**b**) A grey-level histogram of the GFRP sample.

The significant density contrast between glass fibers (2.45–2.54 g/cm^3^) and the epoxy matrix (1.1–1.25 g/cm^3^) enables distinct phase separation. During scanning, 2D projections acquired over a 360° rotation were reconstructed into a 3D model [41,42]. The resulting voxel grayscale values, which are directly proportional to the linear attenuation coefficient *μ*, exhibit histogram peaks representing air, resin, and composite [43], as shown in Figure 4b. For quantification, the macroscopic fiber bundle volume fraction (*V*_bundle_) was quantified using the voxel count of fiber bundles (*N*_bundle_) relative to the total volume (*N*_total_):

(2) $$V_{bundle}=\frac{N_{bundle}}{N_{total}}\times 100\%$$

#### 2.2.2. Ignition Loss Method

Given the discrepancies in CT-derived volume fractions, the ignition loss method (ASTM D2584 [44]) was employed to obtain accurate measurements. Samples were heated at 565 °C in a muffle furnace (Yamato Scientific FO511C) to volatilize the matrix, isolating the glass fibers for mass quantification. The relevant equations are presented below, where *M*_GF_ is the glass fiber mass fraction, *M*_After_ is the total sample mass after testing, *M*_Before_ is the total sample mass before testing, *V*_GF_ is the glass fiber volume fraction, *ρ*_GFRP_ is the density of GFRP, and *ρ*_GF_ is the density of glass fiber.

(3) $$M_{GF}=\frac{M_{After}}{M_{Before}}\times 100\%$$

(4) $$V_{GF}=M_{GF}\times \frac{\rho_{GFRP}}{\rho_{GF}}\times 100\%$$

### 2.3. Mechanical Test

All mechanical tests in this study were conducted under the guidance and recommendations of the relevant ASTM or ISO standards. To ensure experimental reproducibility, the tests were performed under standard ambient laboratory conditions at a room temperature of 23 ± 2 °C and a relative humidity of 50 ± 10%. Furthermore, prior to testing, the width and thickness of each specimen were measured three times at the mid-section using a calibrated digital micrometer or vernier caliper. The average values of these measurements were then used to determine the accurate initial cross-sectional area for subsequent stress calculations.

#### 2.3.1. Tensile Test

**Figure 5:**
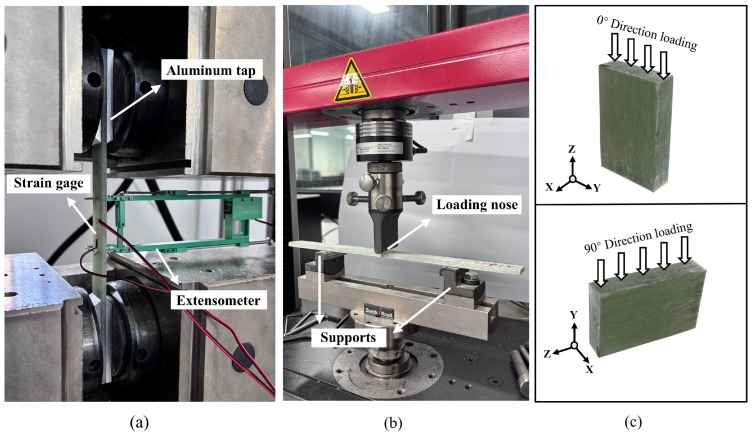
(**a**) Tensile test setup of GFRP sample; (**b**) Three-point flexural test setup of GFRP sample; (**c**) Loading direction of compression test.

The tensile test of GFRP followed ASTM D3039 [45] using a WANCE WDYA305 electro-hydraulic servo machine (Shenzhen Wance Testing Machine Co., Ltd., Shenzhen, China) at a loading speed of 2 mm/min (Figure 5a). Samples were reinforced with aluminum end-tabs (80 mm × 25 mm × 6 mm) to prevent gripping damage. An extensometer measured axial elongation, with the Elastic Modulus calculated within the 0.001–0.003 strain range. Poisson’s ratio was determined using transverse strain gauges.

#### 2.3.2. Flexural Test

Three-point bending tests were conducted per ASTM D7264 [46] using a Zwick Z010 machine (ZwickRoell, Ulm, Germany) at 1.0 mm/min (Figure 5b). Samples (300 mm × 25 mm × 5 mm) were tested with a 160 mm support span (32:1 span-to-thickness ratio) to mitigate shear failure. Flexural stress (*σ*) and strain (*ε*) were calculated using the following equations, where *F* is the applied load, *l* is the span, *b* is the sample width, *h* is the sample thickness, and *z* is the measured deflection.

(5) $$\sigma =\frac{3Fl}{4bh^{2}},\;\;\;\;\;\varepsilon =\frac{6zh}{l^{2}}$$

#### 2.3.3. Compression Test

The compression test referenced ASTM D695 [47] and ISO 14126 [48] using a Lishi 300 kN machine (Shanghai Lishi Scientific Instrument Co., Ltd., Shanghai, China) at 2 mm/min. As shown in Figure 5c, the Z-axis is the fiber direction, and the cuboid samples were tested in two orientations: the 0° direction corresponds to loading parallel to the fibers, while the 90° direction corresponds to loading perpendicular to the fibers. The specimens were prepared as short cuboid blocks with dimensions of 36 mm (length) × 24 mm (width) × 6 mm (thickness) and tested in an end-bearing configuration directly between two flat parallel steel platens. Specifically, in the 0° longitudinal compression, the loading height was 36 mm, resulting in a low height-to-thickness ratio of 6.0. In the 90° transverse compression, the loading height was 24 mm, yielding an even lower ratio of 4.0. Due to the very small height and extremely low slenderness ratio of the machined specimens, specialized anti-buckling fixtures were not required. This specific geometric provision inherently provides sufficient structural stability to effectively minimize the risk of macroscopic global buckling prior to ultimate compressive failure.

#### 2.3.4. Shear Test

**Figure 6:**
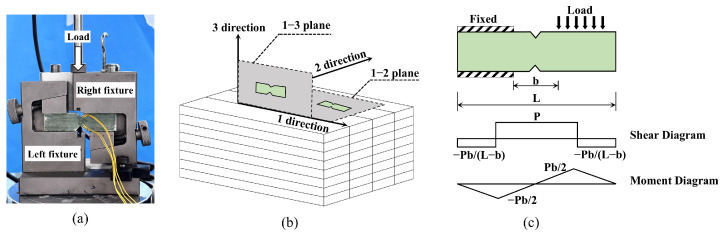
V-notch shear test configuration. (**a**) V-notch fixture; (**b**) Samples extraction planes; (**c**) Shear and moment diagrams.

Given the anisotropy of composite materials, the shear properties exhibit appreciable dependence on both the loading direction and the stress state. Thus, the GFRP shear test items included interlaminar shear (out-of-plane shear) strength, short beam strength, and in-plane shear strength, all of which were evaluated using an Instron 5967 machine (Instron, Norwood, MA, USA) with a 30 kN capacity. Interlaminar and in-plane shear tests followed ASTM D5379 [49] using a standard V-notch fixture (Figure 6). Interlaminar samples (1–3 plane) assessed interfacial bonding, while in-plane samples (1–2 plane) measured shear perpendicular to the layup. Shear strain (*γ*) was calculated from two ±45° strain gauges using Equation (6).

(6) $$\gamma =\left|\varepsilon_{{+45}^{{}^{\circ}}}\right|+\left|\varepsilon_{{-45}^{{}^{\circ}}}\right|$$

The Short Beam Shear (SBS) strength was determined per ASTM D2344 [50] to assess apparent strength under complex stress states [51]. To prevent premature flexural failure in these thick-section composites during shear testing, a test span of 32 mm and a span-to-thickness ratio of 2:1 were adopted. This ratio was strictly maintained for all specimens. Shear strength (*τ*_sbs_) was calculated based on the peak load *P_m_* and sample geometry, where *b* and *h* are the measured specimen width and thickness, respectively, and *y* is the distance between the interlaminar crack and the neutral axis.

(7) $$\tau_{sbs}=0.75\times \frac{P_{m}}{b\times h}\times \left[1-{\left(\frac{2y}{h}\right)}^{2}\right]$$

## 3. Results and Discussion

### 3.1. Microstructural Characterization of GFRP

**Figure 7:**
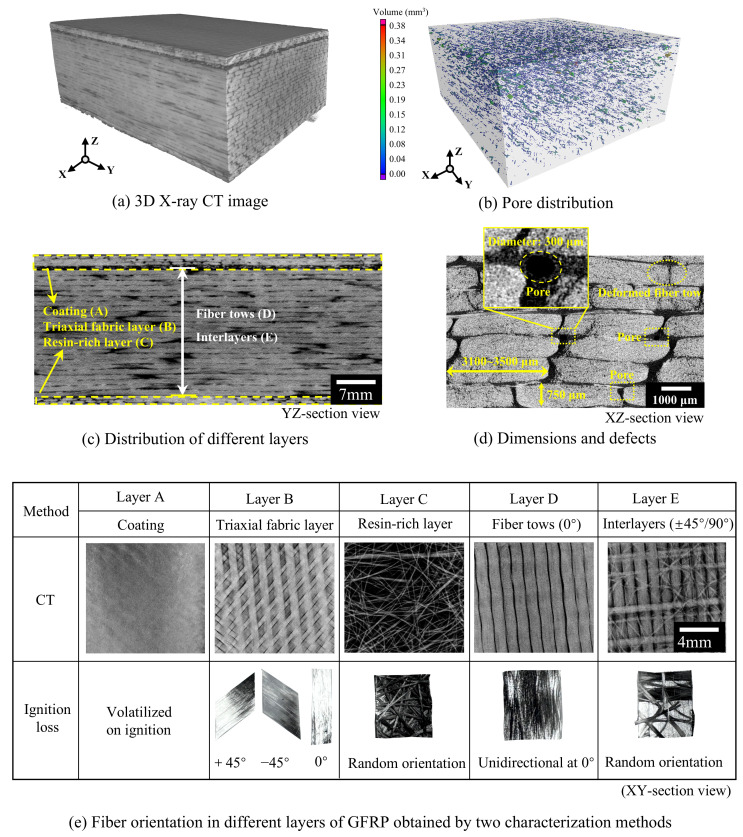
X-ray CT image of a typical GFRP sample (The minimum voxel size: 7.3 μm).

Figure 7 presents the 3D rendering and cross-sectional details. The blade consists of a layered composite structure. Specifically, the mechanical analysis focuses on the unidirectional fiber layers D and E, which constitute the majority of the thickness. As shown in Figure 7d, layer E uniquely contains 90° oriented fiber tows between the main tows, which constrain the 0° fibers effectively during tensile loading, thus dominating the mechanical response of GFRP. Figure 7d shows that fiber tows (approx. 3100–3500 μm wide, 750 μm thick) exhibit irregular geometries due to local compression and internal splaying. Near-circular features (approx. 300 μm diameter) identified in Figure 7d are manufacturing-induced pores. As determined by volumetric CT analysis in Figure 7b, these discrete pores are uniformly distributed and account for a low overall porosity of only 1.2%, which is well below the typical 2% acceptable threshold [52] for high-quality composites. Furthermore, no pervasive fatigue-induced interfacial micro-cracking or fiber-matrix debonding was observed around these voids. This confirms that the pores originated from manufacturing rather than accumulating during the 10-year service, indicating the absence of severe service-induced volumetric damage and the preservation of its meso-structural architecture.

The numerical difference between the two methods arises from the scale limitations of X-ray CT and the physical definition of the segmented phases. As shown in Table 1, the volume fractions measured by micro-CT for samples in the three regions are 89.34%, 87.75%, and 84.27%, respectively. These values represent the macroscopic fiber bundle volume fraction (*V*_bundle_) rather than the absolute pure glass fiber volume fraction. This distinction is caused by the Partial Volume Effect (PVE). The voxel resolution of the current CT scan is 42 μm, whereas the true diameter of a single glass fiber is approximately 9–13 μm. Because the microstructural features are smaller than the scanning resolution, a single voxel inevitably encapsulates a mixture of individual fibers and the intra-tow microscopic resin gaps. Although achieving sub-micron resolution via nano-CT could physically isolate individual fibers, it necessitates reducing the sample to a millimeter scale. This reduction compromises the macroscopic Representative Elementary Volume (REV) required for evaluating the mechanical performance of the GFRP. It should be noted that this resolution was specifically selected for fiber volume fraction quantification, differing from the imaging parameters employed in Figure 7 to characterize the ply layup.

Consequently, the threshold segmentation mathematically isolates the mesoscopic fiber bundles as a unified phase. To evaluate the material’s internal architecture, a multi-scale approach is applied by calculating the intra-tow local fiber volume fraction (*V*_f,local_). This parameter is expressed as the ratio of the pure glass fiber volume fraction (*V*_GF_), derived from the IL test, to the macroscopic fiber bundle volume fraction (*V*_bundle_) obtained from CT, as shown in Equation (8). Taking the sample with an IL result of 61.41% and a CT result of 89.34% as an example, the calculated *V*_f,local_ is approximately 68.7%. This value is comparable to the intra-tow fiber volume fractions (68.3–69.7%) reported in recent literature [53]. While not as precise as direct micro-scale measurements, this agreement suggests that the proposed macro-to-micro derivation provides a reasonable estimation, adequately capturing the mesoscopic compaction state of the material.

(8) $$V_{f,local}=\frac{V_{GF}}{V_{bundle}}\times 100\%$$

By removing the resin matrix, the ignition loss test enabled a layer-by-layer dissection to visually corroborate the fiber architecture observed in the CT scans, as shown in Figure 7e. To accurately interpret the mechanical data and the spatial property variations, the function and structure of each distinct layer are explicitly defined based on these combined microstructural characterizations:

(1) Layer A (Surface Coating/Gel Coat): Completely volatilized during the ignition loss test, this layer acts as the protective topcoat for the blade.
(2) Layer B (Triaxial Fabric Layer): As revealed by the ignition loss results, it features an angular sequence of [+45°/0°/−45°]. This layup is specifically designed to distribute multi-directional loads and enhance the shear strength of the outer aerodynamic shell.
(3) Layer C (Resin-rich Layer/Surface Mat): Corresponding to the randomly oriented fibers observed post-ignition, this layer is typically a chopped strand mat used for surface finish improvement or as an internal bonding layer.
(4) Layers D and E (Structural Core Layers): These layers constitute the primary load-bearing section, composed of alternating unidirectional (UD) fiber tows (0°, Layer D) and multiaxial/random interlayers (+45°/90°, Layer E). This repeating [0°/Interlayer] architecture is a classic characteristic of wind blade spar caps.

Consequently, the complete through-thickness architecture of the investigated GFRP laminate, incorporating both structural and non-structural components, can be explicitly expressed as: [Gelcoat/(+45°/0°/−45°)/Mat/(0°/±45°/90°)_n_].

### 3.2. Mechanical Analysis and Failure Mechanisms

#### 3.2.1. Load-Displacement Responses and Stress State Mechanisms

**Figure 8:**
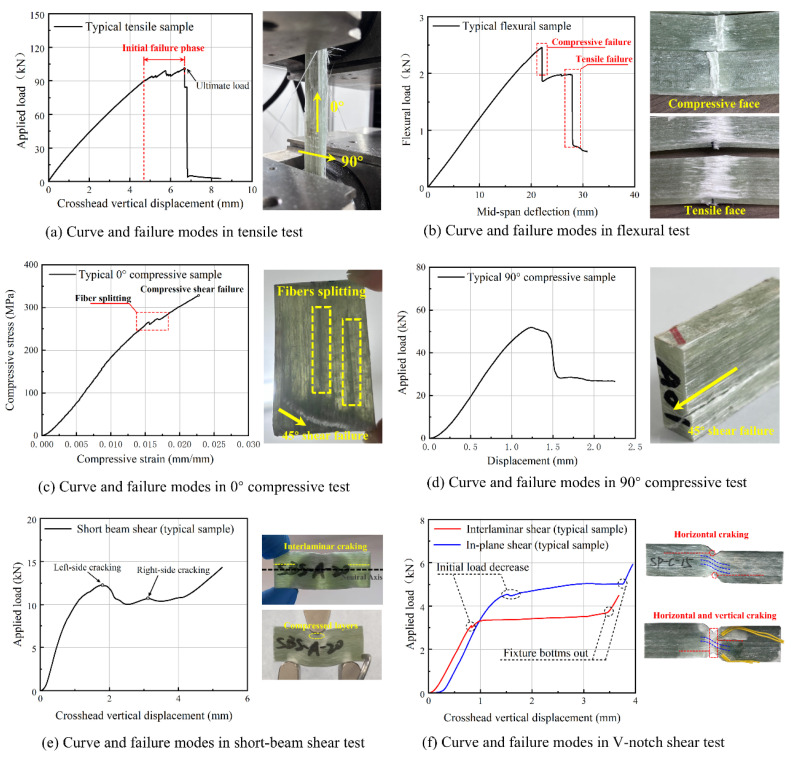
Typical load–displacement curve and failure modes of GFRP under different loadings.

The load-displacement curves and corresponding failure modes directly reflect the transition from fiber-dominated to matrix/interface-dominated mechanisms under different stress states. Under longitudinal tension, the curve in Figure 8a is characterized by a macroscopically linear elastic ascent, confirming a predominantly fiber-dominated mechanism where the continuous glass fibers uniformly carry the load. The localized sawtooth phase (e.g., 4.76–6.68 mm deformation) observed prior to the peak load corresponds to edge-initiated fiber tow debonding. Upon reaching the ultimate tensile capacity, the precipitous load drop marks a catastrophic brittle fracture, governed by instantaneous tow fracture, interlaminar decoupling, and extensive brush-like fiber pull-out.

In contrast, matrix- and interface-dominated loading modes exhibit distinctly different progressive failure behaviors. For flexural tests, the large span-to-thickness ratio (32:1) effectively suppresses the interference of shear effects, ensuring that specimen failure is dominated by bending stresses, thereby emphasizing the role of bending stresses and resulting in a two-stage progressive failure: initial compressive crushing on the upper surface, followed by catastrophic tensile fiber fracture on the lower surface, as shown in Figure 8b. For compression tests, as observed in Figure 8c,d, the profound anisotropy of the GFRP is evident: 0° compression demonstrates fiber splitting and crushing (fiber-dominated stability), whereas 90° compression forces a cooperative load-bearing between the matrix and fibers, yielding a penetrating shear plane with tow sliding. Similarly, the distinct initial load drops in the V-notch and short-beam shear (SBS) curves, as shown in Figure 8e,f, clearly signify the onset of matrix cracking and interlaminar crack propagation in the vicinity of the neutral axis.

#### 3.2.2. Relationship Between Glass Fiber Volume Fraction and Mechanical Strength

The mechanical properties are summarized in Table 2, Table 3 and Table 4. The macroscopic strength of the blade exhibits a spatial dependence on the fiber volume fraction. As determined by the rigorous ignition loss tests, the true volume fraction steadily decreases from 61.41% at the root to 57.21% at the tip. According to the classical rule of mixtures (ROM) [54] in composite mechanics, longitudinal tensile strength is highly fiber-dependent. This volumetric gradient provides a quantitative explanation for the significant spatial drop in ultimate tensile strength from the root (849.5 MPa) to the tip (747.6 MPa). Conversely, the interlaminar shear strength (ILSS) and transverse compressive strength, which are governed predominantly by the polymer matrix and the interfacial bonding quality rather than the absolute fiber volume, exhibit minimal spatial variance along the 54-m blade span.

#### 3.2.3. Effect of Manufacturing Defects and Service Aging

The microstructural characterization provides a fundamental rationale for the observed performance retentions. High-resolution X-ray CT revealed near-circular manufacturing-induced pores of approximately 300 μm in diameter, as illustrated in Figure 7d. From a fracture mechanics perspective, the influence of these discrete pores is highly dependent on the loading mode. Under longitudinal tension, these resin pores do not sever the continuous load-bearing fiber tows; thus, they fail to act as primary crack initiators, allowing the root section to maintain an exceptional high residual tensile strength of 849.5 MPa. However, under matrix-dominated transverse compression and interlaminar shear, these pores act as critical stress concentrators, facilitating localized matrix yielding, tow sliding, and the initial load-drops observed in the shear curves.

Remarkably, despite a 10-year operational history, the GFRP exhibits a void content of only 1.2%, with no detectable interfacial cracking in the high-resolution CT scans. Widespread service-induced mechanical damage within the resin matrix was not observed. This preservation of microstructural integrity can likely be attributed to the fact that the 2.0 MW blade was decommissioned proactively due to technical repowering, rather than reaching its ultimate fatigue life limit. Furthermore, the tested specimens were extracted from the interior of the thick spar cap, which physically shielded the core material from direct severe environmental exposure. This macroscopic shielding, combined with the low initial manufacturing porosity, explicitly justifies the superior residual mechanical properties of these megawatt-scale composites compared to legacy end-of-life blades.

#### 3.2.4. Residual Tensile Strength Verification

To verify the physical rationality of the relatively high tensile strength (849.5 MPa) measured in the primary load-bearing region at the root, a theoretical evaluation was conducted based on the ROM [54]. First, it should be clarified that the test specimens corresponding to this strength value were all strictly cut parallel to the principal fiber direction from a predominantly unidirectional (UD) laminate region, ensuring the accurate measurement of a pure longitudinal tensile response. According to the simplified ROM for unidirectional composites, assuming the load-bearing contribution of the matrix at the moment of tensile fracture is negligible, the longitudinal tensile strength of the composite, *σ*_c_, is predominantly governed by the fibers, as expressed in Equation (9).

(9) $$\sigma_{c}\approx \sigma_{f}V_{GF}$$

where *σ*_c_ is the longitudinal tensile strength of the composite; *σ*_f_ is the effective tensile strength of the fibers and *V*_GF_ is the fiber volume fraction.

Based on the fiber volume fraction of *V*_GF_ = 61.41% measured via the ignition loss method, the back-calculated effective tensile strength of the glass fibers (*σ*_f_) in this region is approximately 1383.33 MPa. Considering that this blade was manufactured between 2015 and 2016, the Chinese National Standard effective at that time (GB/T 18369-2008) [55] stipulated that the minimum initial nominal tensile strength for E-glass fibers used in pultrusion or winding processes must reach 1600 MPa. The calculated results indicate that after 10 years of service and environmental aging, the fibers still retain approximately 86.5% of their initial standard strength. This strength retention rate falls well within a reasonable physical evolution range, demonstrating that the macroscopic tensile strength test results are accurate. The relatively high value is a reasonable consequence of the exceptionally high fiber content (greater than 60%) specific to the spar cap region of large-scale wind turbine blades, combined with a relatively short decommissioning age (10 years). This finding further substantiates that such high-quality decommissioned GFRP materials possess immense structural repurposing potential for high-load-bearing offshore structures, such as wave energy converters.

### 3.3. Multi-Dimensional Comparison of Blade Performance

**Figure 9:**
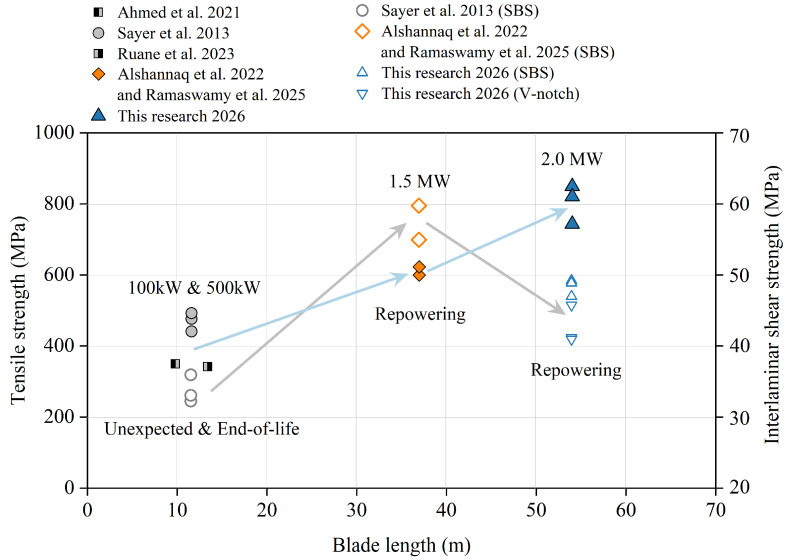
Tensile and shear property variation of GFRP along blade length (with unit capacity). (solid dots: tensile strength; hollow dots: shear strength). Ahmed et al. [30], Sayer et al. [29], Ruane et al. [24], Alshannaq et al. [32], Ramaswamy et al. [33].

Table 5 demonstrates a significant monotonic positive correlation between GFRP tensile strength and blade scale (capacity and length). Conversely, Figure 9 reveals a non-monotonic trend in ILSS: due to manufacturing challenges in ultra-thick sections (e.g., micro-defects from exotherm control), the ILSS of the 2 MW blade is slightly lower than that of the medium-scale (1.5 MW) blade. Nevertheless, analysis of decommissioning reasons indicates that 2 MW blades, retired for repowering after short service lives, retain exceptional mechanical integrity compared to End-of-Life legacy blades. Consequently, despite the minor compromise in shear performance, the superior retained tensile strength and stiffness constitute the core competitiveness of the 2 MW blade as a high-value resource for structural reuse (e.g., in civil engineering or marine engineering applications).

### 3.4. Statistical Analysis of GFRP Mechanical Properties

To analyze the variation in the mechanical properties of GFRP across different blade regions after service and to determine representative statistical values for secondary structural design, a two-stage statistical approach will be employed. First, an Analysis of Variance (ANOVA) was applied, utilizing the blade region as the independent variable to assess intergroup differences. Second, the two-parameter Weibull distribution characterized the statistical properties of GFRP and derived characteristic values reflecting material reliability.

#### 3.4.1. GFRP Properties Variance Analysis

**Figure 10:**
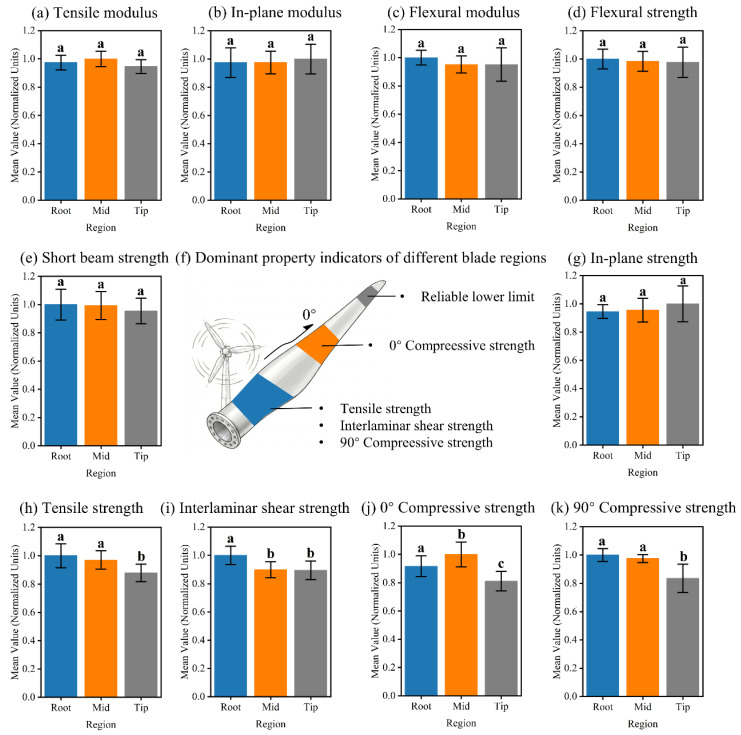
ANOVA results summary of mechanical properties for GFRP blades across different regions. Different lowercase letters (a, b, c) above the bars indicate statistically significant differences (*p* < 0.05). To account for data distribution characteristics, significance was determined using standard One-way ANOVA for (**a**–**e**) and (**g**); Welch’s ANOVA for (**c**) and (**k**); and the non-parametric Kruskal-Wallis H test for (**d**,**i**,**j**).

The GFRP samples were sourced from key sections of decommissioned turbine blades, reflecting potential property variations due to service loads and manufacturing processes. To rigorously evaluate these variations across blade sections, a robust statistical framework was employed. Depending on the outcomes of the Shapiro-Wilk (normality) and Levene (homogeneity of variance) tests, differentiated methods were applied: standard One-way ANOVA with Tukey’s HSD test for ideal data; Welch’s ANOVA coupled with the Games-Howell test for heteroscedastic data. In such cases, if any single group within a specific property violated the normality assumption, the non-parametric Kruskal-Wallis H test, followed by Holm-corrected pairwise Mann-Whitney U tests, was uniformly applied across all three sections [56]. Statistical significance was intuitively visualized via the compact letter display method (Figure 10). Notably, stiffness-governed properties (tensile, flexural, and in-plane shear moduli) and specific shear/flexural strengths exhibited statistical homogeneity (*p* > 0.05, Figure 10a–e,g). This consistency, primarily governed by intrinsic fiber-matrix properties within the small-strain elastic range, justifies pooling these datasets to enhance statistical reliability.

Conversely, significant spatial heterogeneity (*p* < 0.05) emerged in fiber-dominated strengths (tensile, 0°/90° compressive) and interface-dependent ILSS (Figure 10h–k), with the tip section showing notably lower performance. This spatial disparity reflects the blade’s original structural design: during service, the root withstands combined loads (aerodynamic, gravitational, and centrifugal), necessitating high strength, whereas the tip experiences the lowest loads, permitting the use of lower-strength materials for weight reduction. Consequently, the tip region’s lower glass fiber volume fraction, coupled with non-uniform stress distribution, results in reduced reinforcement efficiency. This structural inefficiency ultimately contributes to the observed poorer tensile and compressive properties.

Based on this distinct spatial mapping, a target-oriented graded utilization strategy is proposed (Figure 10f): (i) the Root, excelling in tensile, ILSS, and 90° compressive strengths, is prioritized for critical connection members; (ii) the Mid-span, maximizing 0° compressive strength, is optimal for axial compression members; and (iii) the Tip, maintaining normalized indices above 0.8, serves as a reliable property lower bound for general reuse.

#### 3.4.2. GFRP Properties Characteristic Value Analysis

Based on the grouping results from the preceding variance analysis, where material properties were either categorized by specific regions or merged where no significant difference existed, characteristic values were calculated for each identified dataset. To ensure statistical rigor suitable for structural design, the calculation followed the ASTM D7290 standard [57]. Unlike the traditional normal distribution, this standard employs a two-parameter Weibull distribution, which effectively captures the strength variability of composite materials in the low-probability region—critical for characterizing brittle fracture and weakest-link failure mechanisms [58]. The standard specifies the characteristic value (*X*_char_) as the lower bound of the 80% confidence interval for the population 5th percentile. The probability density function and the characteristic value are calculated as follows:

(10) $$X_{char}=\Omega X_{0.05}$$

(11) $$f\left(x\right)=\left(\frac{\beta }{\alpha }\right){\left(\frac{x}{\alpha }\right)}^{\beta -1}exp\left[-{\left(\frac{x}{\alpha }\right)}^{\beta }\right]$$

To contextualize these results within established design frameworks, this study adopts the comparative assessment method utilized by Alshannaq et al. [31]. The derived ASTM D7290 characteristic values were benchmarked against traditional normal distribution limits (typically μ–2σ and μ–3σ). As presented in Table 6, the Weibull characteristic values for the 2 MW blade consistently fall between these two bounds, providing a stable safety margin that is more conservative than the mean-based limit but less punitive than the 2σ criterion. This observation aligns perfectly with Alshannaq’s findings on 37 m (1.5 MW) blades. Crucially, explicitly extending this validation to the 54 m (2 MW) blade in this study demonstrates that the reliability of the ASTM D7290 method is independent of blade scale. Despite the significant increase in blade size and structural complexity, the Weibull-based characteristic value remains a robust and consistent indicator for structural design.

## 4. A Case Proposal for Repurposing: A Raft-Type Wave Energy Converter

### 4.1. Application Background and Advantages

**Figure 11:**
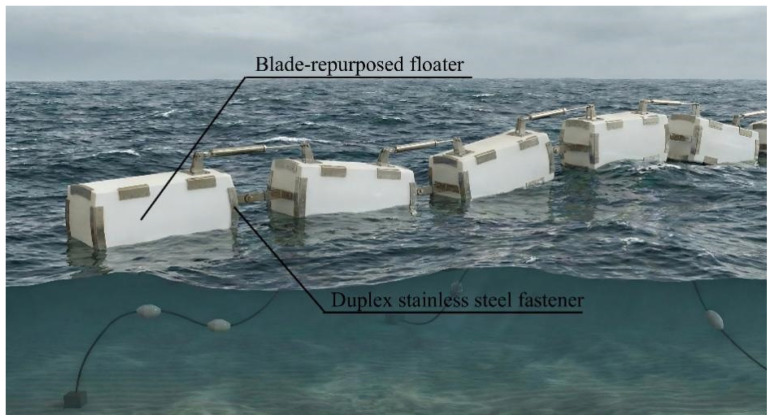
Conceptual diagram of a blade-repurposed raft-type wave energy converter.

Building on the superior residual mechanical properties verified in Section 3, a raft-type wave energy converter (WEC) is proposed to valorize decommissioned wind turbine blades (Figure 11). The design utilizes spliced GFRP blade plates as the primary load-bearing shell, effectively substituting traditional marine-grade steel. Crucially, the repurposed WEC is designed to largely inherit the functional configuration and hydrodynamic parameters of the original steel device. By essentially functioning as a skin replacement and utilizing counterweights to match the original center of gravity, this strategy bypasses the need for complex structural redesigns and significantly lowers the barrier to practical engineering application. To ensure structural integrity in corrosive marine environments, a hybrid connection system employing duplex stainless steel (DSS) profile plates and blind bolts, augmented by structural adhesive for watertight sealing, is adopted.

The rationale for this material substitution is grounded in the environmental hotspots of current wave energy technologies. Existing Life Cycle Assessment (LCA) studies of the WEC consistently identify the material acquisition and manufacturing stage as the dominant contributor to the total carbon footprint [59,60]. This high impact is intrinsically linked to the massive mass of steel structures and the carbon-intensive nature of the steel industry. Consequently, replacing the steel shell with repurposed blade waste offers an opportunity to directly mitigate the most carbon-intensive stage of the WEC life cycle. To quantify this potential, a comparative carbon footprint assessment is conducted in the following section.

### 4.2. Comparative Carbon Footprint Assessment

#### 4.2.1. Methodology of Simplified Carbon Footprint Assessment

To quantify the decarbonization potential of repurposing DWTBs as the structural floating body of a raft-type WEC, a comparative carbon footprint assessment between the blade-repurposed WEC and a conventional Steel WEC is conducted following the principle of ISO 14040 [61]. The assessment focuses exclusively on the Global Warming Potential (GWP), expressed in kgCO_2_ equivalent.

The comparative framework relies on the principle of differential analysis. Since the hydrodynamic geometry and buoyancy performance are designed to be equivalent for both the Steel WEC and blade-repurposed WEC, the following subsystems and life cycle stages are excluded from the boundary as invariant parameters. The Power Take-Off (PTO) system, internal generators, and grid connection infrastructure are identical in both scenarios. The deployment procedures and mooring configurations are functionally equivalent for floating bodies of similar displacement. It must be explicitly stated that the current evaluation is a simplified comparative carbon assessment rather than a comprehensive LCA in accordance with ISO 14040 standards. The system boundary is strictly limited to the material acquisition, transportation and floating body manufacturing stages, with the installation, maintenance and end-of-life stages excluded due to their equivalent carbon emission contributions for the steel-based and GFRP-based WECs with identical hydrodynamic and functional configurations. While GFRP offers superior corrosion resistance, the operation and maintenance stage is excluded. Therefore, the system boundary is strictly limited to three stages:

(1) Material acquisition;
(2) Transportation;
(3) Floating body manufacturing.

The functional unit is defined as a 20.0 m length, 4.0 m diameter WEC segment. Adhering to DNV-RP-C202 [62], the Steel WEC employs a 1.4 stiffener coefficient on a 16-mm shell, yielding a total mass of 44.2 tons. Conversely, the blade-repurposed WEC utilizes 50-mm decommissioned blade laminates with DSS connectors and adhesive, optimizing the mass to 27.65 tons. Under the cut-off approach, blades are treated as zero-carbon resources, while emission factors for steel, adhesive, and electricity are based on the Stainless Steel Branch of CISA, Ecoinvent 3.8, and Chinese national averages, respectively. To ensure accuracy, a two-stage logistics model distinguishes between the volume-limited transport of raw blades (400 km, 0.72 kgCO_2_/t·km) and the standard weight-limited transport of finished modules (200 km, 0.12 kgCO_2_/t·km). The inventory parameters are shown in Table 7.

#### 4.2.2. Results and Discussion of Simplified Carbon Footprint Assessment

The comparative life cycle assessment demonstrates a distinct environmental advantage for the blade-repurposed WEC (Blade WEC) over the Steel WEC. Detailed results summarized in Table 8 indicate that the blade-repurposed solution achieves a total carbon footprint of 29.9 tons of CO_2_, representing a substantial 71.5% reduction compared to the 104.8 tons baseline of the Steel WEC. Concurrently, the structural mass decreases by 37.4% (from 44.2 to 27.65 tons). These results validate that the proposed waste valorization strategy is both a viable waste management solution and an effective pathway for decarbonizing marine infrastructure.

The primary driver of this decarbonization is the substitution of carbon-intensive marine steel. Although the integration of duplex stainless steel connectors and epoxy adhesives introduces approximately 22 tons of carbon emissions, this increase is significantly outweighed by the avoided emissions from primary steel production. Furthermore, while the volume-limited nature of transporting hollow blade segments results in transportation emissions nearly five times higher than those in the steel scenario, this logistical burden accounts for less than 10% of the total carbon savings, confirming the environmental justification for long-distance blade waste transport.

**Figure 12:**
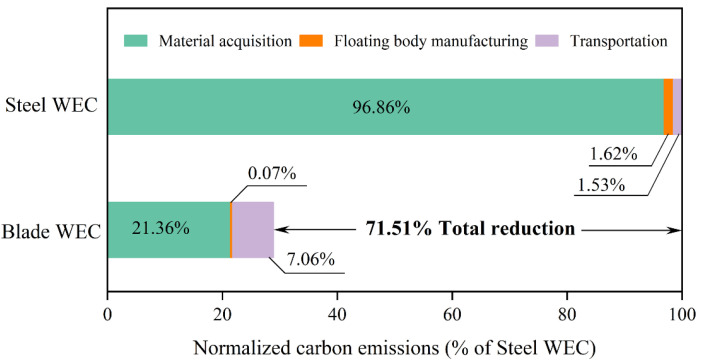
Carbon emission analysis results of the blade-repurposed and the steel WEC.

Crucially, a conservative approach is adopted in the manufacturing assessment to ensure robustness. For the Steel WEC, only direct energy inputs for welding and rolling are included, while energy-intensive processes such as sandblasting, anti-corrosion coating, and facility overheads are excluded. Thus, the estimated emissions represent a theoretical lower bound. Despite this conservative baseline, the blade-repurposed WEC still achieves a 71.5% emission reduction, as shown in Figure 12, highlighting the inherent superiority of low-energy mechanical processing over steel’s thermal manufacturing. In practical industrial scenarios with mandatory corrosion protection, its decarbonization potential is projected to exceed 80%.

Beyond environmental metrics, the potential for significant weight could offer substantial engineering co-benefits. A 37.4% reduction in structural mass would lower hydrodynamic inertia and decrease tension demands on mooring systems, thus potentially improving device survivability under extreme sea conditions. Furthermore, the inherent corrosion resistance of GFRP may eliminate the need for toxic antifouling coatings, which could provide an additional environmental benefit by avoiding the release of heavy metals and microplastics into the marine ecosystem.

## 5. Conclusions

This study addresses the critical data gap concerning the reuse potential of large-scale (>50 m) wind turbine blades by comprehensively characterizing a 54-m (2.0 MW) GFRP blade decommissioned due to repowering. The following key conclusions are drawn:

1. Unlike legacy blades retired at their end-of-life, this 10-year-old repowered blade exhibited minimal internal voids (~1.2%) without observable interfacial debonding and retained exceptional mechanical performance. X-ray CT and ignition loss tests confirmed that observed micro-defects were manufacturing-induced rather than service-related damage. Mechanical results revealed that the root section retained a tensile strength of 849.5 MPa, significantly surpassing reported values for smaller (<1.5 MW) baseline blades. This provides the first empirical evidence that large-scale blades retired for technical upgrades serve as high-quality structural resources rather than mere waste.
2. ANOVA results revealed significant spatial heterogeneity in strength properties, necessitating a location-specific repurposing approach. A graded reuse strategy is proposed: root sections, with superior tensile and shear capabilities, are designated for critical load-bearing members; mid-span sections, exhibiting peak compressive strength, are optimal for axial compression components; and tip sections serve as a reliable baseline for general utility. The characteristic values derived via ASTM D7290 and Weibull analysis further verify that these materials meet the statistical reliability required for structural engineering design.
3. The proposed raft-type Wave Energy Converter (WEC) demonstrates the practical viability of this material substitution. By replacing conventional marine steel with repurposed GFRP laminates, the blade-repurposed WEC achieves a 37.4% reduction in structural mass and a 71.5% reduction in carbon footprint. This confirms that repurposing DWTBs is not only mechanically feasible but also offers a substantial environmental advantage for decarbonizing the marine renewable energy sector.

While this study establishes a robust baseline for the structural repurposing of 2.0 MW blades, several important limitations must be acknowledged. First, the current findings are derived from a single representative 54-m blade and evaluated exclusively under static loading conditions. Second, there is an absence of chemical and thermal matrix characterizations (such as differential scanning calorimetry (DSC), dynamic mechanical analysis (DMA), or Fourier-transform infrared spectroscopy (FTIR)), which are necessary for a comprehensive evaluation of polymer degradation at the molecular level. To advance widespread engineering adoption, future research must prioritize long-term durability assessments under extreme environmental conditions, as well as investigate DWTB GFRP joining strategies for robust connection designs. Furthermore, while this current study focuses exclusively on evaluating the fundamental macroscopic mechanical properties of the decommissioned GFRP material, validating the full-scale performance of the proposed WEC is essential. Therefore, comprehensive structural finite element analysis (FEA), hydrodynamic load evaluations, and subsequent wave pool experiments are currently underway and will be systematically presented in future work. Finally, developing rapid non-destructive evaluation (NDE) technologies—integrating portable CT and machine learning—will enable on-site assessment and optimize the recycling value chain.

## Figures and Tables

**Table 1:** Summary of ignition test results of GFRP.

Blade Region	*V*_GF_ by Ignition Loss (%)			*V*_bundle_ via X-Ray CT (%)			*V*_f,local_ (%)		
	Mean	SD	CV	Mean	SD	CV	Mean	SD	CV
Root	61.41	0.86	0.55	89.34	0.52	0.58	68.74%	0.80%	1.17%
Mid	58.45	0.74	1.13	87.75	0.44	0.50	66.61%	1.12%	1.68%
Tip	57.21	2.00	0.31	84.27	0.99	1.18	67.90%	2.95%	4.34%

*V*_GF_: glass fiber volume fraction; *V*_bundle_: macroscopic fiber bundle volume fraction *V*_f,local_: Intra-tow local fiber volume fraction (*V*_GF_/*V*_bundle_);; SD: standard deviation; CV: coefficient of variation, *ρ*_GF_ = 2.54 g/cm^3^ represents the nominal density of the E-glass fiber used for the calculation of *V*_GF_.

**Table 2:** Summary of mechanical property results from GFRP tensile and flexural tests.

Items	Blade Region	Strength			Modulus		
		Mean (MPa)	CV (%)	Num	Mean (GPa)	CV (%)	Num
Tensile Properties	Root	849.50	8.38	10	42.70	5.26	12
	Mid	824.37	6.58	10	43.86	5.45	13
	Tip	747.64	6.96	10	41.48	5.17	8
Flexural Properties	Root	872.06	6.96	14	39.31	5.24	14
	Mid	857.37	7.19	14	37.42	6.37	14
	Tip	852.42	10.72	12	37.41	12.39	12

**Table 3:** Summary of mechanical property results from GFRP compression tests.

Blade Region	90° Compressive Strength			0° Compressive Strength		
	Mean (MPa)	CV (%)	Num	Mean (MPa)	CV (%)	Num
Root	197.19	4.46	12	476.54	7.98	10
Mid	192.26	2.83	12	519.98	8.75	13
Tip	164.86	11.94	12	422.16	8.60	13

**Table 4:** Summary of GFRP shear property results.

Blade Region	Interlaminar Shear Strength	In-Plane Shear Strength	Short Beam Shear Strength	In-Plane Shear Modulus
	Mean ± SD (Num) (MPa)			Mean ± SD (Num) (GPa)
Root	45.86 ± 2.92 (16)	71.06 ± 3.69 (12)	49.26 ± 5.39 (15)	4.09 ± 0.44 (10)
Mid	41.24 ± 2.62 (16)	71.84 ± 6.34 (12)	48.93 ± 4.89 (15)	4.09 ± 0.34 (10)
Tip	41.06 ± 3.03 (16)	75.18 ± 6.70 (10)	47.04 ± 4.46 (15)	4.20 ± 0.44 (10)

**Table 5:** Comparison of tensile test data of DWTBs with literature.

Research		This Research	Ramaswamy et al. [33]	Alshannaq et al. [32]	Ruane et al. [24]	Sayer et al. [29]	Ahmed et al. [30]
Installed Capacity		2 MW	1.5 MW	1.5 MW	500 KW	100 KW	100 KW
Service Duration		10 years	11 years	11 years	27 years	18 years	20 years
Blade Length		54 m	37 m	37 m	13.4 m	11.6 m	9.8 m
Tensile Strength (MPa)	Root	849.50	-	597	342	476.6	-
	Mid	820.37	620	-	-	492.6	350
	Tip	743.52	-	-	-	441.1	-
Tensile Modulus (GPa)	Root	42.70	-	36.8	27.5	26.7	-
	Mid	43.86	37.5	-	-	26.9	15.6
	Tip	41.48	-	-	-	24.3	-

**Table 6:** Three types of characteristic values.

Item	Blade Region	μ–2σ (MPa)	μ–3σ (MPa)	*X*_char_ (MPa)
Tensile Strength	Root	707.1	635.9	642.9
	Mid	715.9	661.6	661.8
	Tip	643.6	591.6	588.1
Tensile Modulus	All	39.4 × 103	38.2 × 103	38.3 × 103
Flexural Strength	All	821.6	795.2	800.0
Flexural Modulus	All	31.7 × 103	28.5 × 103	30.3 × 103
0° Compressive Strength	Root	400.5	362.5	371.0
	Mid	428.9	383.4	378.9
	Tip	349.5	313.2	335.7
90° Compressive Strength	Root	179.6	170.8	171.7
	Mid	181.4	175.9	174.5
	Tip	125.5	105.8	114.0
Interlaminar Shear Strength	Root	40.0	37.1	37.2
	Mid	36.0	33.4	34.5
	Tip	35.0	32.0	34.7
In-plane Shear Strength	All	67.2	65.6	64.8
In-plane Shear Modulus	All	3.8 × 103	3.7 × 103	3.6 × 103
Short Beam Shear Strength	All	40.2	35.9	38.9

**Table 7:** Carbon emission analysis inventory parameters.

Parameter	Steel WEC	Blade WEC	
Total mass	44.19 tons	27.65 tons	
Transport distance	300 km	400 km	200 km
Transport factor	0.12 kgCO_2_/t/km	0.72 kgCO_2_/t/km	0.12 kgCO_2_/t/km
Material emission factor	2.30 kgCO_2_/kg	0 (blades); 5.5 (duplex stainless steel); 8.1(adhesive)	
Grid emission factor	0.5703 kgCO_2_/kWh	0.5703 kgCO_2_/kWh	
Manufacturing energy	3000 kWh	120 kWh	

**Table 8:** Results of carbon footprint assessment from two types of WECs at different stages.

Items	Material Acquisition (kgCO_2_)	Transportation (kgCO_2_)	Floating Body Manufacturing (kgCO_2_)
Steel WEC	101,632	1591	1710
Blade WEC	22,416	7411	68

## Data Availability

The original contributions presented in this study are included in the article. Further inquiries can be directed to the corresponding author.

## Abbreviations

The following abbreviations are used in this manuscript:

EOL	End-of-Life
GFRP	Glass Fiber-reinforced Polymers
GHG	Greenhouse Gas
CT	Computed Tomography
ROM	Rule of Mixtures
IL	Ignition Loss
SBS	Short Beam Shear
PVE	Partial Volume Effect
REV	Representative Elementary Volume
DSS	Duplex Stainless Steel
LCA	Life Cycle Assessment
GWP	Global Warming Potential
PTO	Power Take-Off
NDE	Non-Destructive Evaluation
